# HIV infection, hypercoagulability and ischaemic stroke in adults at the University Teaching Hospital in Zambia: a case control study

**DOI:** 10.1186/s12879-017-2455-0

**Published:** 2017-05-18

**Authors:** Stanley Zimba, Patrice Mukomena Ntanda, Shabir Lakhi, Masharip Atadzhanov

**Affiliations:** Department of Internal Medicine, University of Zambia, P.O.Box 51237, Lusaka, Zambia

**Keywords:** Sub-Saharan Africa, Zambia, Hypercoagulability, Ischaemic stroke, HIV infection

## Abstract

**Background:**

In Zambia, 14.2% of adults have HIV/AIDS. There has been a substantial and significant increase in patients hospitalized for ischaemic stroke with co-existing HIV infection. However, little is known about the mechanism of stroke in these HIV + ve patients let alone studied in our region. The aim of this pilot study was to explore the association of hypercoagulability state in HIV + ve patients with ischaemic stroke. This was achieved by comparing hypercoagulability state markers between HIV + ve ischaemic stroke patients with HIV-ve and HIV + ve patients with and without ischaemic stroke respectively.

**Methods:**

A matched case control study in which a total of 52 HIV + ve patients with ischaemic stroke were prospectively compared with control groups for the presence of protein S, protein C deficiencies and hyperhomocysteinaemia. The control groups comprised an equal number of consecutively matched for age and sex HIV-ve and HIV + ve patients with and without ischaemic stroke respectively. Data was analysed in contingency tables using Paired t- test, Chi square and conditional logistic regression.

**Results:**

Ischaemic stroke of undetermined aetiology occurred more frequently in HIV + ve compared to HIV-ve patients (*p* < 0.001). In addition, protein S deficiency and Hyperhomocysteinaemia were more prominent in HIV + ve than HIV-ve ischaemic stroke patients (*P* = 0.011). There was no difference in the presence of hyperhomocysteinaemia or protein S deficiency in HIV + ve patients with or without ischaemic stroke. Protein C deficiency was not noted to be significantly different between the cases and the two control arms.

**Conclusion:**

Protein S deficiency and hyperhomocysteinaemia were associated with HIV infection, but not stroke in our study population. However, this is an area that requires extensive research and one that we cannot afford to ignore as it is an important bridge to all cardiovascular and cerebrovascular diseases.

## Background

In Zambia, 14.2% of adults have Human Immuno-deficiency Virus/Acquired Immuno-deficiency syndrome (HIV/AIDS) [1]. There has been a substantial and significant increase in patients hospitalized for ischaemic stroke with co-existing HIV infection [2–6]. This is rapidly becoming a serious problem and is significantly adding to the rising burden of non-communicable diseases [7–9]. We cannot afford to ignore such an observation as it is compounding the infectious and poverty related disease burden and it is further straining the limited resources channelled to the health sector [10, 11].

Little is known about the mechanism of ischaemic stroke in these HIV positive (HIV + ve) patients as few studies have been done in our region. Possible mechanisms of stroke in HIV infection include vascular abnormalities, coagulopathies and cardio-embolic disease from clinical and pathological studies from Europe and Latin America [12]. Coagulopathies could occur by variable mechanisms, but more so possibly by the presence of protein S (PS) deficiency, protein C (PC) deficiency, and hyperhomocysteinaemia as a result of the HIV infection mediated chronic inflammation [13, 14].

The purpose of this pilot study was to explore the association of hypercoagulability state with ischaemic stroke in adult Zambian HIV + ve patients. This was achieved by comparing hypercoagulability state markers between HIV + ve ischaemic stroke patients with HIV-ve and HIV + ve patients with and without ischaemic stroke respectively. By including the HIV + ve patients without ischaemic stroke, this helped in determining whether hypercoagulability state was associated with HIV infection or stoke.

## Methods

The study design was a matched case control study of adults aged 18 years and above at the University Teaching Hospital (UTH) in Lusaka, Zambia. The target population was adult patients seen at UTH either as in-patients or out-patients via Adult Medical Emergency Unit (AMEU), Clinic five, Adult Infectious Diseases Centre (AIDC), and Physiotherapy departments.

A total of 52 HIV + ve patients with ischaemic stroke were prospectively compared with control groups for occurrence of PS, PC deficiencies and hyperhomocysteinaemia. The definitions of PS, PC deficiencies and hyperhomocysteinaemia were as recommended by the International Federation of Clinical Chemistry (IFCC) [15]. The control groups comprised an equal number of consecutive matched for race, sex and age at +/− 5 years interval HIV negative (HIV-ve) and HIV + ve patients with and without ischaemic stroke respectively. Ischaemic stroke was confirmed by clinical assessment and brain imaging [computer tomography (CT) scan or magnetic resonance (MRI)]. Patient recruitment was done after the acute phase of stroke with stroke duration more than 48 h, but up to 1 month and the recruitment period was July 2014 to February 2015.

Hypertension was defined in this study as current use of antihypertensive medication, history of being diagnosed as hypertensive by a doctor prior to stroke, documented blood pressure of greater than or equal to 140 mmHg systolic or 90 mmHg diastolic before the stroke or persisting more than a week after the acute event (World Health Organization) or evidence of left ventricular hypertrophy on ECG or Echocardiography.

Diabetes mellitus was diagnosed if a patient was taking anti-diabetic drugs prior to stroke; if a doctor had diagnosed type I or type II diabetes before stroke; if a patient had a documented non fasting blood glucose of greater than 11.1 mmol/L, or a fasting blood glucose of greater than or equal to 7.0 mmol/L after the acute phase of stroke.

Cigarette smoking was classified as smoker (current or former smoker for more than 1 year) or non smoker, and alcohol consumption as non drinker or drinker (ex-drinker for more than 1 year or current alcohol use).

Laboratory investigations such as Full Blood Count (FBC) measured using FBC SYSMEX 2000 and 4000, CD_4_ count measured using FACS Calibur, Liver Function Tests (LFTs), cholesterol, urea and creatinine measured using Beckman Coulter AU480 were done at UTH as part of all patient routine work up. The HIV antibody tests were done on all recruited patients using a combination of Determine and Bioline test kits, with the Unigold kit as a tie-breaker. For PS, PC deficiencies and hyperhomocysteinaemia, venous blood sample was collected and immediately transported on ice at a temperature of 2 – 8 Degrees Celsius to a private lab (Lancet-Nkanza Laboratory) for separation of plasma and cellular components before the samples were flown to Lancet Laboratory in Johannesburg, South Africa, for analysis using Hemosil™ immunoassays.

The examination included a detailed neurological assessment with measurement of blood pressure, and assessment for any evidence of vascular disease including hypertensive end-organ damage affecting the heart or fundal vessels and any potential sources of emboli. Assessment for signs of focal neurological deficit (hemiparesis, hemi-sensory loss, cranial nerves palsy, aphasia, level of consciousness, alexia, agraphia, and apraxia) was done together with the patient’s National Institutes of Health Stroke Scale (NIHSS) at the time of examination. Patients were reviewed with all data collection sheets, scans and other investigations, and assigned a final diagnosis plus stroke type using the Trial of Org 10,172 in acute stroke treatment (TOAST) classification. Instances where no cause of ischaemic stroke was found because some investigations had not been done before the patient died or the patient had no funds to pay for some investigations which were not covered by the study were classified as incomplete evaluation under TOAST classification [16, 17].

Patients with haemorrhagic stroke, Sickle Cell Disease (SCD), no neuro-imaging or focal neurological deficit of non-vascular origin were excluded from the study. In addition, taking anticoagulant drugs, contraceptive pills, hormone replacement therapy (HRT), pregnancy, liver disease and refusal to do HIV test were part of the exclusion criteria.

The sample size was calculated by first making the assumption that 19% of all HIV + ve ischaemic strokes were due to coagulopathies as reported by Tipping et al. [18] Then, the occurrence of coagulopathies in the general population was estimated at 4% taking into account Remkova’s postulation that hereditary deficiencies of Antithrombin III, PC or PS can be found in fewer than 5% of unselected patients [19]. The confidence interval was set at 95% and the power to detect a difference at 80%. There were three arms with the case being HIV + ve ischaemic stroke and two controls – one control being HIV-ve ischaemic stroke and the other being HIV + ve no stroke. A total of 51 subjects were required in each of the three arms.

All the data was entered and analysed on Statistical Package for the Social Sciences (SPSS) statistics 2012 version 21. Data were analysed in contingency tables using Paired t test for means and McNemar’s Chi square test for proportions. Conditional logistic regression step-down model was used to measure for association. The variables for logistic regression were selected using biological plausibility on the basis of association with hypercoagulability state and also variables with significant *p* values after comparison between cases and controls. For the conditional logistic regression analysis of HIV + ve compared to HIV-ve ischaemic stroke, the selected variables included age, hypertension, obesity, hyperhomocysteinaemia, PS deficiency, smoking, previous ischaemic stroke/TIA and major risk factor for stroke. For ischaemic stroke compared to no stroke HIV + ve patients, PC deficiency, age, alcohol, ART use, hypertension and Diabetes Mellitus were the variables used for step-down analysis. A *p*-value of less than 0.05 was taken as the level of statistical significance.

Ethical approval was obtained from ERES Converge International Research Board (IRB) (ref. No. 2013 – Dec – 009) and permission was obtained from UTH management to conduct the study. Written informed consent to participate in the study as well as for publication was obtained from each of the study participants. In the event that the participant had altered mental status, informed consent was obtained from the surrogate who was a care-giver and close relative of the participant.

## Results

**Fig. 1:**
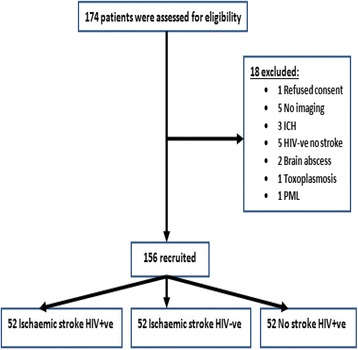
Recruitment process

One hundred and seventy-four patients were approached as potential candidates for inclusion into the study. Of these, 18 patients were excluded from the study with ten patients either HIV-ve with no stroke or had no brain imaging. Of the remainder, one patient refused consent and seven patients had neurological findings other than ischaemic stroke. One hundred and fifty-six patients were recruited of which 52 patients were HIV + ve and had ischaemic stroke. These patients were frequency matched for age and sex with 52 HIV-ve ischaemic stroke patients and 52 HIV + ve no stroke patients as controls respectively. (Fig. 1).

The demographic and clinical findings of ischaemic stroke patients by HIV status are shown in Table 1 and described briefly here. Despite attempts to match by age range (+/− 5 years), there was a statistical difference between the two groups and as expected the HIV-ve ischaemic stroke patients tended to be older. Eight patients in the ischaemic stroke HIV-ve arm had previous ischaemic stroke compared to none in the HIV + ve group. Of the traditional risk factors, hypertension, smoking and obesity were found to be statistically significant and they were noted to be more prominent in the HIV-ve ischaemic stroke patients.

**Table 1:** Comparison of baseline demographic and clinical characteristics between HIV + ve and HIV-ve ischaemic stroke patients

Characteristics	Ischaemic stroke HIV + ve *N* = 52	Ischaemic stroke HIV-ve *N* = 52	*P* – value
Age, years, mean (Std. Dev.)	52 (13.0)	61 (11.5)	**<0.001** ^a^
Sex, female, n (%)	29 (56)	29 (56)	0.999
Hypertension, n (%)	26 (50)	39 (75)	**0.008** ^**a**^
Diabetes mellitus, n(%)	8 (15)	6 (12)	0.566
Smoking, n(%)	2 (4)	11 (21)	**0.008** ^**a**^
Alcohol, n (%)	10 (19)	6 (12)	0.277
Obesity, n(%)	2 (4)	11 (21)	**0.008** ^**a**^
Previous ischaemic stroke/TIA, n(%)	0 (0)	8 (15)	**0.003** ^**a**^
Family history of stroke, n (%) Major risk factors for stroke, n (%)	13 (25) 44 (85)	13 (25) 51 (98)	0.999 **0.016** ^**a**^
NIHSS score at enroll, n (range)	8 (2 – 15)	11 (4 – 19)	0.254
Oxford handicap at enroll, mean(%)	6 (12)	2 (4)	0.269
Hypercholesterolaemia > 5.2 mmol/l	4 (8)	7 (13)	0.999
Hb, g/dl (range)	12.6 (10.5–14.6)	12.8 (10.7–15.0)	0.599
Serum creatinine(>120umol/l)n(%)	6 (17)	7 (23)	0.498

^a^statistically significant

**Fig. 2:**
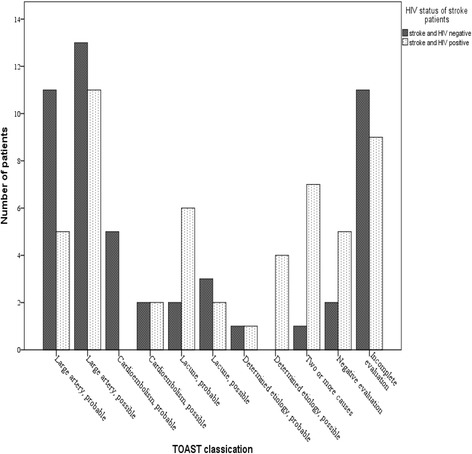
TOAST classification comparing ischaemic stroke by HIV status

The subtypes of ischaemic stroke using TOAST classification were compared between the HIV + ve and HIV-ve patients using a bar chart (Fig. 2). Seventeen patients in the HIV + ve ischaemic stroke arm compared to four in the HIV-ve arm had stroke of undetermined aetiology either due to negative evaluation or due to the presence of two or more causes. The difference between the two was statistically significant (*p* < 0.001). Nine patients in the HIV + ve ischaemic stroke arm had stroke of undetermined aetiology due to incomplete evaluation compared to 11 patients in the other arm and the difference was not statistically significant (*p* = 0.310). In addition, the HIV-ve arm had most of its patients with large artery atherosclerosis compared to the HIV + ve arm (*p* = 0.050).

Table 2 shows the demographic and clinical characteristics of the ischaemic stroke HIV + ve patients now compared to no stroke HIV + ve patients. Despite frequency matching by age range (+/− 5 years), there was a noted statistical difference between these two groups. The majority of the patients in the HIV + ve ischaemic stroke arm where on antiretroviral therapy (ART) compared to the no stroke arm. The same could be said for hypertension as there was a statistically significant difference between the two arms.

**Table 2:** Comparison of baseline demographic and clinical characteristics between ischaemic stroke and no stroke HIV + ve patients

Characteristics	Ischaemic stroke HIV + ve *N* = 52	No stroke HIV + ve *N* = 52	*P* - value
Age, years, mean(Std. Dev.)	52 (13.0)	46 (11.8)	**0.014** ^a^
Sex, female, n(%)	29 (56)	29 (56)	0.999
Hypertension, n(%)	26 (50)	9 (17)	**<0.001** ^a^
Diabetes mellitus, n(%)	8 (15)	3 (6)	0.111
Alcohol, n(%)	10 (19)	4 (8)	0.085
Smoking, n(%)	2 (4)	2 (4)	0.999
ART use(current & previous), n(%)	36 (69)	46 (88)	**0.016** ^a^
NRTI use, n(%)	34 (65)	49 (94)	0.999
NNRTI use, n(%)	33 (63)	46 (88)	0.641
PI use, n(%)	1 (2)	3 (6)	0.644
ART duration(>3 months), n(%)	47 (90)	51 (94)	0.205
Hypercholesterolaemia > 5.2 mmol/l	4 (8)	4 (8)	0.999
Serum creatinine(>120umol/l),n(%)	6 (12)	4 (8)	0.322
Hb, g/dl (range)	12.6 (10.5 – 14.6)	12.2 (9.7 – 14.7)	0.470
CD_4_ count, cells/ul (range)	431 (111 – 751)	422 (164 – 680)	0.885

^a^statistically significant

Table 3 illustrates the comparison of hypercoagulability state markers between the cases (ischaemic stroke HIV + ve) and the two controls (ischaemic stroke HIV-ve and no stroke HIV + ve). Hyperhomocysteinaemia and PS deficiency were significantly present in the cases compared to the two controls. In addition, smoking, obesity and sedentary lifestyle were statistically found in the ischaemic stroke HIV-ve control compared to the cases and the no stroke HIV + ve control.

**Table 3:** Comparison of hypercoagulability state markers between the three arms (cases and two controls)

Variables	Ischaemic stroke HIV + ve *N* = 52	Ischaemic stroke HIV-ve *N* = 52	No stroke HIV + ve *N* = 52	Stroke HIV + ve vs. stroke HIV-ve *P*-value	Stroke HIV + ve vs. No stroke HIV + ve *P*-value
Hyperhomocysteinaemia > 14.4umol/l	33 (63)	20 (38)	27 (52)	**0.011** ^**a**^	0.234
Protein S deficiency < 60%	22 (42)	10 (19)	18 (35)	**0.011** ^**a**^	0.420
Protein C deficiency < 70%	5 (10)	5 (10)	0 (0)	0.999	0.057

^a^statistically significant

Tables 4 and 5 show significant variables after step down binary logistic regression analysis. The first table shows the variables for HIV + ve ischaemic stroke compared to HIV-ve ischaemic stroke patients and the second table shows HIV + ve ischaemic stroke compared to HIV + ve no stroke patients. This was done to assess for the association of hypercoagulability state markers and traditional risk factors with ischaemic stroke in HIV + ve patients. Protein S deficiency, Protein C deficiency and hyperhomocysteinaemia were not associated with Ischaemic stroke or HIV infection on logistic regression analysis. Obesity and age were negatively associated with ischaemic stroke in HIV + ve individuals whereas smoking had a positive association in these patients. ART use and hypertension were, however, noted to be associated with ischaemic stroke among HIV infected individuals. Alcohol intake was noted to be protective against ischaemic stroke although we did not accurately quantify the amounts taken by our patients.

**Table 4:** Factors associated with the probability of HIV infection among patients with ischemicstroke. Logistic regression analysis

Variables	Crude OR (95% CI)	Adjusted OR (95% CI)	*p*–value (Adj. OR)
Age	0.94 (0.91 – 0.97)	0.94 (0.90 – 0.98)	0.001
Smoking	6.70 (1.41 – 31.99)	6.08 (1.19 – 30.95)	0.030
Obesity	0.15 (0.03 – 0.71)	0.16 (0.03 – 0.80)	0.026

**Table 5:** Factors associated with the probability of ischemic stroke among HIV-infected individuals. Logistic regression analysis

Variables	Crude OR (95% CI)	Adjusted OR (95% CI)	*P*-value (Adj. OR)
Hypertension	4.78 (1.94 – 11.76)	4.51 (1.51 – 13.47)	0.007
Alcohol	0.35 (0.10 – 1.20)	0.15 (0.03 – 0.69)	0.015
ART use	5.43 (0.61 – 48.16)	8.77 (0.91 – 84.65)	0.061

## Discussion

We analyzed the subtypes of ischaemic stroke by HIV status on the basis of aetiology using TOAST classification. We found that 33% of the HIV + ve ischaemic stroke patients presented with stroke of undetermined aetiology due to negative evaluation compared to 8% in the HIV-ve group. The HIV-ve group had large artery atherosclerosis as the most important aetiological mechanism accounting for 46% of its patients whereas it only accounted for 30% in the HIV + ve group. This can be explained by Ortiz et al. [12] who found that the incidence of vasculitis and hypercoagulability state is relatively high in HIV + ve patients and a likely mechanism for ischaemic stroke occurrence in these patients.

We compared variables that promote a hypercoagulability state between the HIV + ve and the HIV-ve ischaemic stroke patients, and an additional HIV + ve with no stroke patients. PC deficiency did not show any significant difference between these groups although hyperhomocysteinaemia and PS deficiency were significantly more in the HIV + ve ischaemic stroke group compared to the HIV-ve ischaemic stroke group. Homocysteine levels are elevated in vitamin B_12_ and folic acid deficiency which is a common finding in HIV + ve patients who present with micronutrient deficiencies. Hence the reason why there was no noted difference in the presence of hyperhomocysteinaemia between the HIV + ve ischaemic stroke and HIV + ve with no stroke groups. This argument would have been strengthened if the body mass index (BMI) of all the patients we recruited had been done and compared as well as actual documentation serum B_12_ levels. Nonetheless, hyperhomocysteinaemia has been strongly linked to progression of generalized small-vessel disease as was evidenced by Kloppenborg et al. who found that a role existed for homocysteine in the development of a generalized small-vessel disease in the brain [20]. Jeon et al., in looking at homocysteine, small-vessel disease and atherosclerosis also concluded that hyperhomocysteinaemia was associated with small-vessel disease of the brain and large-vessel disease of cerebral arteries [21].

On the contrary, the study by Coria-Ramirez et al. [22] added a new dimension to this discussion. They studied the effect of combination anti-retroviral therapy (cART) on homocysteine plasma concentrations in HIV-1 infected patients. They found that fasting and post-oral methionine load plasma homocysteine levels increased after 6 months of anti-retroviral treatment. Furthermore, they concluded that nutritional abnormalities were not responsible for hyperhomocysteinaemia, but suggested the enzymatic disturbances in the metabolic pathways of homocysteine that occurred after initiation of cART. The evidence from this study is worth taking into account considering 48% of the HIV + ve ischaemic stroke patients had been on cART for more than 3 months at the time of enrolment into our study. On logistic regression analysis, however, we found that hyperhomocysteinaemia was not significantly associated with HIV infection or ischaemic stroke. This was an unexpected finding which needs further verification with a higher powered study, a larger sample size and also focusing on the role of genetic polymorphism particularly those related to homocysteine.

With regards to PS deficiency, we did not find any significant association with HIV infection as was elicited in many studies including the Mochan et al. study in South Africa. In addition, we did not find any association with HIV + ve ischaemic stroke [23–25]. PS is produced by the liver and endothelial cells, and acts as a cofactor to activated PC in the inactivation of factor Va and factor VIIIa. The mechanism by which PS deficiency occurs is not fully understood, but is thought to result from the presence of autoimmune antibodies or because of inflammatory markers such as interleukin 1, cytokines and tumour necrosis factor alpha stimulated by HIV [12].

The association of hypercoagulability state with ischaemic stroke in HIV infection is definitely a complex mechanism with many factors at play. Other anticoagulation factors that may have a role in this hypercoagulability state and need to be evaluated include Anithrombin III, factor V Leiden, lupus antibodies, anticardiolipin antibodies and prothrombin G20210A mutation [13]. In addition, genetic factors as well as polymorphisms are an important area of interest and serve as a limitation to our study. Moreover, there is evidence from twin and family-based studies which seems to suggest a substantial heritability for ischaemic stroke with different associations by ischaemic stroke subtypes [26–29]. This is exemplified by a large observational study in the United States of America which found that coagulation Factor XIII B-subunit contributed to risk of ischaemic stroke of cardioembolic subtype [30]. In this study, the HIV status of the participants was unknown and it is not entirely correct for these findings to be generalised to HIV + ve ischaemic stroke patients.

What is clear in our findings, however, is that HIV + ve adults without ischaemic stroke had a similar prevalence of hypercoagulability state markers as HIV + ve adults with ischaemic stroke. This is a significant point against the idea that these markers cause stroke in HIV-infected adults.

Our study was limited by the relatively small sample size. The funds were inadequate to allow for detailed investigation of the patients such that factor V Leiden, lupus antibodies, anticardiolipin antibodies and antithrombin III could not be done. Some findings were based on information from the routine care available at the hospital during the study. Failure to do brain imaging rendered some patients not to be included in the study due to technical difficulties. Finally, the BMIs for most patients could not be calculated because of lack of a proper weighing machine for bedridden patients.

## Conclusion

HIV + ve adults with ischaemic stroke had more Protein S deficiency and hyperhomocysteinaemia than HIV-ve adults with ischaemic stroke. However, HIV + ve adults with and without ischaemic stroke had similar levels of Protein S deficiency and hyperhomocysteinaemia. Hypercoagulability state was associated with HIV infection, but not stroke in our study population. However, this is an area that requires extensive research and one that we cannot afford to ignore as it is an important bridge to all cardiovascular and cerebrovascular diseases.

## Abbreviations

ART: Anti-retroviral therapy
BMI: Body mass index
cART: Combination active anti-retroviral therapy
CT scan: Computerised tomography scan
Hb: Haemoglobin
HIV: Human immuno-deficiency virus
HIV + ve: Human immune deficiency virus positive
HIV-ve: Human immune deficiency virus negative
ICH: Intracerebral haemorrhage
IRIS: Immune reconstitution inflammatory syndrome
MRI: Magnetic resonance imaging
NIHSS: National Institute of health Stroke Scale
NNRTI: Non-nucleoside reverse transcriptase inhibitors
NRTI: Nucleotide reverse transcriptase inhibitors
OR: Odd ratio
PC: Protein C
PI: Protease inhibitor
PML: Primary multifocal leukoencephalopathy
PS: Protein S
TIA: Transient ischemic attack
TOAST: Trial of org 10,172 in acute stroke treatment
UTH: University Teaching Hospital
